# Evolution of Cost-Free Resistance under Fluctuating Drug Selection in *Pseudomonas aeruginosa*

**DOI:** 10.1128/mSphere.00158-17

**Published:** 2017-07-19

**Authors:** Anita H. Melnyk, Nicholas McCloskey, Aaron J. Hinz, Jeremy Dettman, Rees Kassen

**Affiliations:** a Department of Biology and Centre for Advanced Research in Environmental Genomics, University of Ottawa, Ottawa, Ontario, Canada; b Ottawa Research and Development Center, Agriculture and Agri-Food Canada, Ottawa, Ontario, Canada; Escola Paulista de Medicina/Universidade Federal de São Paulo

**Keywords:** *Pseudomonas aeruginosa*, antibiotic resistance, experimental evolution, fluctuating selection

## Abstract

Antibiotic resistance evolves rapidly in response to drug selection, but it can also persist at appreciable levels even after the removal of the antibiotic. This suggests that many resistant strains can both be resistant and have high fitness in the absence of antibiotics. To explore the conditions under which high-fitness, resistant strains evolve and the genetic changes responsible, we used a combination of experimental evolution and whole-genome sequencing to track the acquisition of ciprofloxacin resistance in the opportunistic pathogen *Pseudomonas aeruginosa* under conditions of constant and fluctuating antibiotic delivery patterns. We found that high-fitness, resistant strains evolved readily under fluctuating but not constant antibiotic conditions and that their evolution was underlain by a trade-off between resistance and fitness. Whole-genome sequencing of evolved isolates revealed that resistance was gained through mutations in known resistance genes and that second-site mutations generally compensated for costs associated with resistance in the fluctuating treatment, leading to the evolution of cost-free resistance. Our results suggest that current therapies involving intermittent administration of antibiotics are contributing to the maintenance of antibiotic resistance at high levels in clinical settings.

**IMPORTANCE** Antibiotic resistance is a global problem that greatly impacts human health. How resistance persists, even in the absence of antibiotic treatment, is thus a public health problem of utmost importance. In this study, we explored the antibiotic treatment conditions under which cost-free resistance arises, using experimental evolution of the bacterium *Pseudomonas aeruginosa* and the quinolone antibiotic ciprofloxacin. We found that intermittent antibiotic treatment led to the evolution of cost-free resistance and demonstrate that compensatory evolution is the mechanism responsible for cost-free resistance. Our results suggest that discontinuous administration of antibiotic may be contributing to the high levels of antibiotic resistance currently found worldwide.

## Abstract (precis)

Antibiotic resistance is a global problem that greatly impacts human health. How resistance persists, even in the absence of antibiotic treatment, is thus a public health problem of utmost importance. In this study, we explored the antibiotic treatment conditions under which cost-free resistance arises, using experimental evolution of the bacterium *Pseudomonas aeruginosa* and the quinolone antibiotic ciprofloxacin. We found that intermittent antibiotic treatment led to the evolution of cost-free resistance and demonstrate that compensatory evolution is the mechanism responsible for cost-free resistance. Our results suggest that discontinuous administration of antibiotic may be contributing to the high levels of antibiotic resistance currently found worldwide.

## INTRODUCTION

Comparative and epidemiological data suggest that the removal of an antibiotic from widespread use does not always lead to increased antibiotic susceptibility in the population (1–3). Indeed, the rate at which resistance declines is often much lower than expected based on measured costs of resistance (4–6) and resistant strains often persist at appreciable levels long after treatment ceases. This result is unexpected because most resistance mutations are energetically costly (7); an antibiotic-sensitive genotype not paying a cost of resistance should therefore rapidly displace resistance genotypes under no-drug conditions (8–10). The effectiveness of antibiotics is thus being undermined not only by the emergence of antibiotic resistance but also by the unexpectedly long-term persistence of resistance in the absence of drug selection.

Managing the arsenal of useful antibiotics thus depends crucially on understanding how antibiotic resistance came to be and why it is so persistent. We hypothesize that therapies involving repeated dosing with periodic intervals of no antibiotic dosing generate persistent antibiotic resistance by selecting for the evolution of cost-free resistant strains. Antibiotic delivery at the level of the individual patient is best characterized as a series of pulses involving periods of high antibiotic concentrations immediately after administration followed by periods of low concentrations once the antibiotic has been metabolized and removed from the body. Repeated drug dosing therefore generates a regular cycle of strong antibiotic selection followed by periods of relaxed selection where drug concentrations are greatly reduced or absent, a form of intermittent or fluctuating selection. These periods of relaxed selection may be prolonged if regular doses are intentionally or inadvertently skipped. Fluctuating selection can select for broadly adapted generalists (11–14) that in this context both are antibiotic resistant and maintain high fitness under no-drug conditions. Such cost-free resistance genotypes are, we suspect, major contributors to persistent drug resistance. The genetic mechanisms responsible for persistence are often attributed either to genetic coselection due to linkage between the resistance gene(s) and selected loci (15) or to compensatory mutations that alleviate the cost of resistance without compromising the resistance itself (16–21). However, it is also possible that occasional “no-cost” mutations, i.e., those that confer resistance but do not incur a large fitness cost under no-drug conditions, arise in the population and are selectively maintained (22–27). The relative importance of these mechanisms to persistence remains unknown.

Here we test the hypothesis that fluctuating selection leads to the emergence of cost-free resistance genotypes by allowing initially isogenic populations of the opportunistic human pathogen *Pseudomonas aeruginosa* to evolve under conditions of either constant or fluctuating selection imposed by the commonly used fluoroquinolone antibiotic ciprofloxacin. We then used whole-genome sequencing of the evolved genotypes to identify the mutations that were selected in each treatment and an allelic replacement protocol to evaluate the impact of the selected mutations on fitness and resistance. This approach allowed us to evaluate experimentally the genetic mechanisms responsible for cost-free resistance.

Our focus on *P. aeruginosa* stems from its roles as a major cause of hospital infections and as the pathogen most commonly associated with mortality in cystic fibrosis (CF) patients (28, 29). *P. aeruginosa* is known to maintain a high level of intrinsic resistance (30), and treatment with fluoroquinolones has been shown to lead to the rapid emergence of high levels of resistance to this class of drug (31). Moreover, the treatment of lung infections in CF patients with oral fluoroquinolones is often periodic (also termed “intermittent administration”; 29, 32), especially during acute exacerbations where lung function is compromised (33). The standard treatment of exacerbations with ciprofloxacin typically lasts around 14 days (29, 34, 35) and sometimes less to improve patient compliance and quality of life, especially for younger patients (33). During periods of treatment, bacteria decrease in abundance within the lung but are not eradicated (36), implying that the evolution of high-fitness resistance genotypes characteristic of compensatory evolution or the selection of cost-free mutations may be common. Our experiment was designed to capture many of the most salient features of treatment of *P. aeruginosa* infections. In particular, we have chosen drug concentrations that mimic those seen in the sputum of CF patients undergoing fluoroquinolone treatment (37) and have limited the durations of treatments used in our study to approximately the duration of a typical exacerbation treatment. *In vitro* experiments such as ours cannot capture all the relevant selection pressures associated with colonization and persistence *in vivo*. For this reason, our genetic work focuses explicitly on the causes of resistance and its associated costs rather than on additional mutations that putatively impact growth under laboratory conditions. This approach is justified by our previous work (38) showing that the genetic targets associated with resistance to ciprofloxacin *in vitro* are very similar to those observed in the clinic. We provide further evidence to support this claim here.

## RESULTS AND DISCUSSION

We tracked the evolution of resistance and fitness in eight replicate populations of *P. aeruginosa* by serial transfer in liquid medium in each of two environments: supplementation with daily doses of ciprofloxacin (constant treatment [c]) and supplementation with intermittent doses of ciprofloxacin (fluctuating treatment [f] [two 24-h cycles with drug, followed by two 24-h cycles without drug]), for a total of approximately 50 generations of antibiotic selection. At the end of the experiment, we isolated a single colony from each evolved population and assayed (i) the MIC, i.e., the lowest concentration of ciprofloxacin that inhibited growth; (ii) growth rates in both the presence and absence of ciprofloxacin; and (iii) relative levels of fitness in competition with the sensitive ancestral strain under no-drug conditions. Whole-genome sequencing of evolved isolates permitted the identification of the genetic targets of selection. Allelic constructs allowed us to quantify an individual mutation’s contribution to resistance and fitness.

Two aspects of our experimental design warrant further discussion. The first aspect is our choice to focus on single colonies, rather than whole populations, as representative endpoints for our experiment. This choice is justified because our main interest is in how the ecology of drug delivery (fluctuating versus constant) impacts the repeatability of phenotypic and genomic evolution associated with drug resistance. An appropriate experimental design therefore requires that we allow replicate populations to evolve independently under each environmental condition and to identify the most common genotype in each. We selected colonies by plating a sample of the entire population on an agar plate and choosing the colony closest to an arbitrary point in the middle of the plate, a technique that ensures that colony selection is random with respect to phenotype or morphology. The selected colony is thus expected to have been among the most abundant genotypes in the population, though there is always a small possibility that it was not. Reassuringly, we found a positive correlation between single isolates and the whole population from which they were isolated for both MIC (see Fig. S1 in the supplemental material; *r* = 0.540, *P* = 0.038) and relative fitness (Fig. S2; *r* = 0.462, *P* = 0.083), as expected if the isolate were a random sample from a larger population. Notably, both measures tended to be greater at the population level than at the individual level (for MIC, *W* = 59, *n*_1_ = 8, *n*_2_ = 7, *P* = 0.022; for fitness, *t* = 5.43, *df* = 14, *P* < 0.001), suggesting that additional diversity exists in these populations. This result is not unexpected, as the short duration of our experiment means that selection may not have had sufficient time to fix weakly beneficial variants (39). While a quantitative description of this diversity is of interest in its own right, it is not required to make strong inferences about the kinds of adaptations that evolve under these different selective conditions and we therefore do not consider it further. The isolate-based genotypic analyses that follow are thus best interpreted as representative examples of a range of possible genetic routes to adaptation in the initial stages following antibiotic treatment.

10.1128/mSphere.00158-17.1 FIG S1  Population MIC as a function of evolved isolate MIC. Download FIG S1, PDF file, 0.1 MB. Copyright © 2017 Melnyk et al. 2017 Melnyk et al. This content is distributed under the terms of the Creative Commons Attribution 4.0 International license.

10.1128/mSphere.00158-17.2 FIG S2  Population relative fitness as a function of evolved isolate relative fitness. Download FIG S2, PDF file, 0.1 MB. Copyright © 2017 Melnyk et al. 2017 Melnyk et al. This content is distributed under the terms of the Creative Commons Attribution 4.0 International license.

The second aspect is the lack of a no-drug control treatment. We chose not to include this treatment because our focus was on the identity and diversity of genetic changes associated with drug resistance specifically rather than on the process of adaptation to no-drug media itself. For this reason, we adjusted our selection regime to equalize the levels of exposure to drug rather than the total amounts of evolution, and we did not include other kinds of controls. Nevertheless, all of the fitness and MIC assays whose descriptions follow were done both in the presence and the absence of drug, to allow direct comparisons of MICs, competitive fitness levels, and growth rates to those of the sensitive, unevolved ancestor from which all isolates in this experiment were derived.

### Fluctuating selection leads to the evolution of cost-free resistance genotypes.

**FIG 1:**
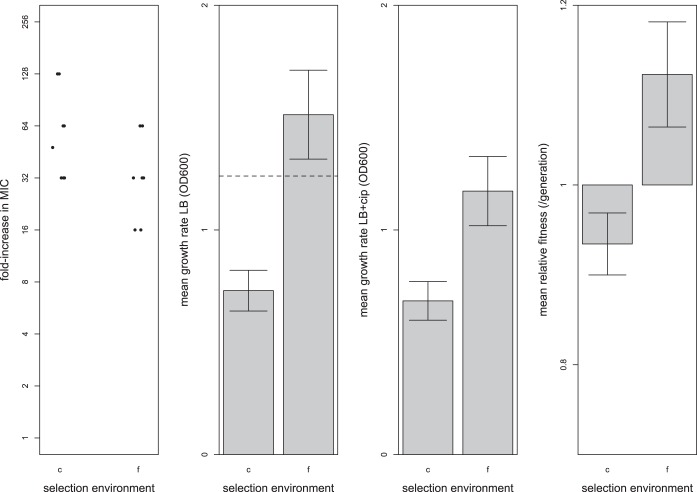
Adaptive responses as measured by the ciprofloxacin MIC (panel 1), mean growth rates (OD_600_) in the absence (panel 2) and presence (panel 3) of ciprofloxacin (cip), and mean relative levels of fitness of evolved isolates in the absence of ciprofloxacin, relative to the ancestor, which has a value of 1 (panel 4). “c” denotes the constant ciprofloxacin treatment, and “f” denotes the fluctuating ciprofloxacin treatment. All error bars represent an SE of ±1. MIC was calculated as the median of the results from four to six trials. Mean growth rate was calculated from three replicate measures for each experimentally evolved isolate. Mean relative fitness was measured in the absence of antibiotic via direct competitions with a *lacZ*-marked ancestral isolate (PA14) using 3 to 10 replicate measures from each experimentally evolved isolate. The dashed line in panel 2 represents the growth rate of the ancestor (the ancestor does not grow in the presence of antibiotic, so no dashed line is provided in panel 3). The relative fitness value of 1 represents the fitness of the ancestor, and a relative fitness value of less than 1 indicates a cost of resistance in the absence of the antibiotic (panel 4).

Selection was effective at generating ciprofloxacin resistance under conditions of both constant treatment and fluctuating treatment, although isolates from the constant treatment had significantly higher MICs than those from fluctuating treatments (Fig. 1, panel 1, *W* = 41.5, *n*_1_ = 8, *n*_2_ = 7, *P* = 0.058). Evolved isolates from the constant treatment grew slower than those from the fluctuating treatment under both sets of conditions (Fig. 1, panel 2, ciprofloxacin absent, *t* = −3.07, *df* = 14, *P* = 0.010; Fig. 1, panel 3, ciprofloxacin present, *t* = −3.97, *df* = 14, *P* < 0.01) and had significantly lower fitness (mean, 0.853, standard error [SE], ±0.0344) than those from the fluctuating treatment (Fig. 1, panel 4) (mean, 1.025, SE, ±0.0585, *t* = −2.78, *df* = 14, *P* = 0.019). These results point to the existence of a trade-off between the level of resistance and fitness measures: high levels of resistance evolving under conditions of constant antibiotic selection come at the cost of lower growth rates and lower fitness in the absence of ciprofloxacin. These results also provide strong support for the hypothesis that the fluctuating drug/no-drug treatments typically experienced by patients on standard antibiotic therapy can select for the evolution of drug-resistant strains that maintain high fitness in the absence of antibiotic.

### Genomics of adaptation.

Whole-genome sequencing of the evolved isolates gave a median coverage of ~38× per genome (mean, 37.6×; range, 28.2× to 49.4×) and revealed between 2 and 5 mutations per isolate (mean, 3.13; SE, ±0.215) compared to the ancestral PA14 genome. The majority of mutations (*n* = 33) were single nucleotide polymorphisms (SNPs), but small insertions (*n* = 2) and deletions (*n* = 12) were also uncovered (Table S1 in the supplemental material lists all mutations and their predicted functional consequences). Together, there were 31 unique changes affecting a total of 14 genes. No large insertions or deletions were found. Not included in the above calculations was a single isolate from the fluctuating treatment that bore a lesion in *mutS* and had 32 mutations, suggesting that it was a mutator strain. Mutators have been shown to gain a fitness advantage when grown under conditions of antibiotic selective pressure *in vivo* (40) and are often observed in clinical settings within the lungs of CF patients (41–44). However, inferences about the genomic targets of selection are complicated by elevated mutation rates, so we did not consider this isolate further.

10.1128/mSphere.00158-17.3 TABLE S1  Mutations detected by Illumina sequencing of evolved isolates. “Sel env” gives the selection treatment as “c” for constant ciprofloxacin and “f” for fluctuating ciprofloxacin. Download TABLE S1, XLSX file, 0.1 MB. Copyright © 2017 Melnyk et al. 2017 Melnyk et al. This content is distributed under the terms of the Creative Commons Attribution 4.0 International license.

Across all replicates, the average number of mutations per genotype under conditions of fluctuating selection (mean, 3.57; SE, ±0.164) was marginally higher than that seen under conditions of constant selection (mean, 2.75; SE, ±0.345; *t* = −2.04, *df* = 14, *P* = 0.074). On a per-generation basis, however, genotypes from the constant treatment contained more mutations (mean, 0.055 mutations/generation) than genotypes from the fluctuating treatment (0.0357 mutations/generation; *t* = 3.91, *df* = 14, *P* < 0.01). This apparently contradictory result came about because we equalized the durations of exposure to antibiotic between the two treatments at ~50 generations, meaning that the fluctuating treatment necessarily evolved for twice as long as the constant treatment. Thus, persistent directional selection imposed by constant ciprofloxacin causes higher rates of genomic evolution.

### Genetic targets of resistance.

**FIG 2:**
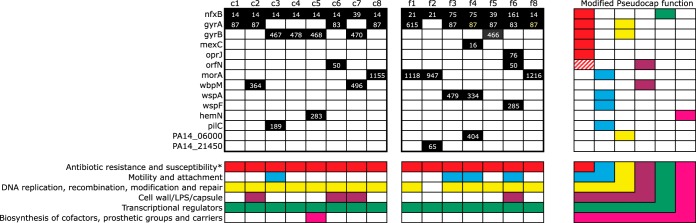
Genes (*n* = 14) containing mutations after selection under conditions of constant or fluctuating ciprofloxacin treatment. The black squares in the large matrix denote genes that had mutational changes, and the numbers within the boxes represent the codon number where the change occurred. Differently colored numbers represent different amino acid changes. The colored squares in the smaller matrices denote PseudoCap (51) functions of the genes. The asterisk indicates that a modified PseudoCap function was used, as known fluoroquinolone resistance-conferring genes were included within this functional category. LPS, lipopolysaccharide.

In *P. aeruginosa*, fluoroquinolone resistance occurs primarily via two main mechanisms (45, 46): (i) mutations to the regulators (*nfxB*, *mexR*) of multidrug efflux pumps (MexCD-OprJ, MexAB-OprM) that act to decrease intracellular fluoroquinolone concentrations (47) and (ii) mutations that prevent fluoroquinolones from binding to DNA-modifying subunits of DNA gyrases (*gyrA*, *gyrB*) or topoisomerases (*parC*, *parE*) (48–50). We found that all isolates carried mutations in the *nfxB* efflux pump regulator and, with the exception of isolate f2, had a second resistance mutation in either *gyrA* or *gyrB* (Fig. 2). These results suggest that mutations in both efflux pump regulators and gyrases may be necessary to confer high levels of resistance and are consistent with previous work showing that combinations of mutations at these two gene targets have additive effects on resistance (51). Mutations in other known fluoroquinolone resistance genes occurred more sporadically; isolate f4 harbored a *mexC* mutation, isolate f6 had an *oprJ* mutation, and isolates c6 and f6 both had mutations in *orfN*, a gene encoding a predicted glycosyl transferase that has previously been shown to confer increased resistance to ciprofloxacin (38). Thus, mutations were found to occur in a range of genetic targets associated with ciprofloxacin resistance in addition to the normal suite of efflux pump regulators and gyrase/topoisomerase targets.

**FIG 3:**
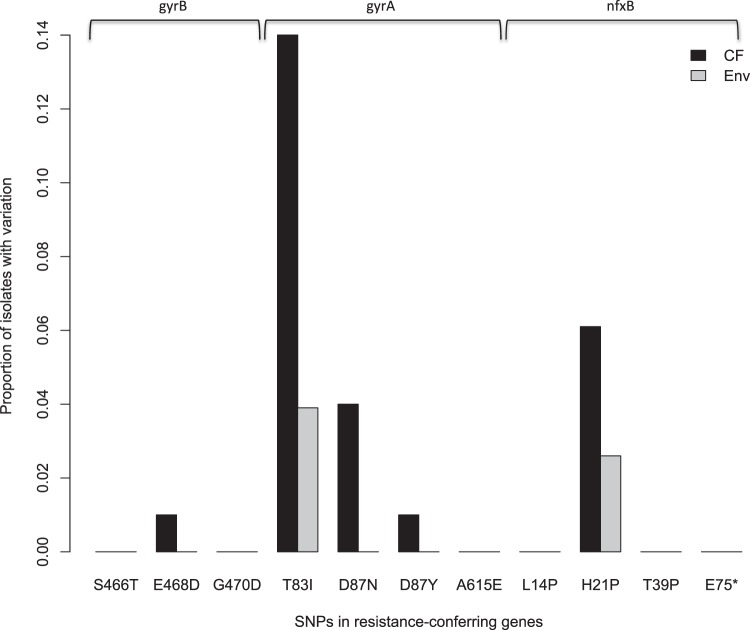
The fraction of isolates with polymorphism present at specific sites within 100 CF isolates (black) and 77 environmental isolates (gray). Shown are the proportions of isolates with variation at the specific base positions found to be mutated in our laboratory selection experiment. We surveyed for variation at the specific base position but have shown codon changes along the *x* axis for ease of interpretation and comparison with other figures presented.

As CF patients are often treated with ciprofloxacin or other fluoroquinolones, we asked whether the genetic targets of ciprofloxacin resistance in our selection experiment were similar to those from *in vivo* isolates from CF patients. We surveyed the sequence variation present within the three canonical resistance genes (*gyrA*, *gyrB*, and *nfxB*) in 100 clinical *P. aeruginosa* isolates sampled from patients with CF and in 77 environmental isolates from various sources that were unlikely to have been exposed to the same high concentrations of ciprofloxacin as those associated with treatment (see Materials and Methods). Strikingly, variation in natural isolates was observed at several of the exact nucleotide positions mutated in our laboratory experiment in a manner consistent with fluoroquinolone-mediated selection among CF isolates (Fig. 3). For example, a high fraction of CF isolates harbored SNPs at nucleotides within codons 83 and 87 of *gyrA*, a result mirrored by the vast majority (90%) of the isolates that had evolved in our experiment. Similarly, we found two isolates with mutations at codon 21 in *nfxB*, another site where there is substantial variation within CF isolates. While there were some notable gaps—codon 14 in *nfxB*, for example, was the most commonly mutated site in this gene in our experiment, but variation was absent in natural isolates—in all cases where a mutation observed in our experiment was also present in natural isolates, its frequency was higher among CF isolates than among environmental ones (Fig. 3). These results suggest that our *in vitro* experiment recapitulates many of the most relevant aspects of selection for resistance among CF patients *in vivo*.

### Second-site mutations are common.

Additional mutations in genetic targets not obviously related to fluoroquinolone resistance, henceforth termed second-site mutations, were observed in nearly all evolved isolates except c1, c4, and f5 (Fig. 2). These second-site mutations were, in fact, nearly as common in the constant environment as they were in the fluctuating environment: 5/8 isolates in the constant treatment compared to 6/7 isolates in the fluctuating treatment, a result that does not represent a statistically significant difference (χ^2^ = 0.184, *df* = 1, *P* = 0.668). The main effect of second-site mutations appears to involve a shift from a motile, planktonic lifestyle to a nonmotile, biofilm-forming state, as evidenced by mutations in *morA*, *wspA*, and *wspF*, genes that are known to affect motility and biofilm formation via cyclic-di-GMP signaling (38, 52–54). Loss of motility was likely adaptive in our experiment because cultures were maintained with constant agitation, meaning that maintenance and assembly of the complex and the energetically costly machinery associated with motility were unnecessary. Other genetic targets included heme biosynthesis (hemN) and formation of type IV pili (*pilC*), both of which harbored frame-shift mutations toward the beginning of the gene that likely produce a truncated protein and so would be expected to result in a loss of function. Taken together, these results suggest that the fitness benefit associated with second-site mutations results primarily from mutations that prevent energy being expended on the expression and maintenance of costly functions not necessary for growth under laboratory conditions.

Several of the most commonly substituted second-site mutations occur in genes that have been identified as genetic targets of selection in CF patients. Marvig and colleagues (41) identified 52 genes that were mutated in multiple CF isolates; these included *wbpM*, *morA*, and *wspA*, along with the canonical resistance mutations in *gyrA*, *gyrB*, and *nfxB*. In a similar survey, Smith and colleagues (55) identified *wspF* mutations in 6 of 29 CF patients surveyed. These results, taken together with those from the investigations of the specific codons associated with ciprofloxacin resistance discussed above, provide good evidence that our experimental conditions captured many of the most salient features associated with drug resistance during adaptation of *P. aeruginosa* to the lungs of CF patients.

### The genetics of cost-free resistance.

It is tempting to interpret the evolution of cost-free resistance under conditions of fluctuating selection as being due to the substitution of second-site mutations that improve fitness but do not compromise resistance. An alternative interpretation is that the evolution of cost-free resistance is attributable to the selection of low-cost resistance alleles in the fluctuating-dose environment and of high-cost ones in the constant-dose environment.

We can distinguish between these alternative hypotheses by comparing the MICs and levels of relative fitness of the evolved genotypes to those of genetic constructs containing specific resistance mutations that arose in our experiment. If the suite of second-site mutations fixed under conditions of fluctuating conditions were compensatory, we would expect that all resistance alleles would be costly under no-drug conditions and that evolved lines (containing the resistance mutations plus all further second-site mutations) would have higher fitness on average than reconstructed genotypes with just the resistance mutations. Alternatively, if fluctuating selection led to the fixation of cost-free resistance mutations, then resistance alleles from the fluctuating treatment would show no evidence of a cost while those from the constant treatment would tend to be more costly. To construct the appropriate genotypes, we used a previously described allelic exchange method (56, 57) to transfer evolved resistance alleles into the ancestral genetic background, either singly or in combination with the other resistance alleles that were fixed in the same genotype. Details on the constructs made are provided in Table S2.

10.1128/mSphere.00158-17.4 TABLE S2  List of allelic constructs assayed in this study, with their respective fold increases in MIC and relative fitness values. Download TABLE S2, XLSX file, 0.1 MB. Copyright © 2017 Melnyk et al. 2017 Melnyk et al. This content is distributed under the terms of the Creative Commons Attribution 4.0 International license.

**FIG 4:**
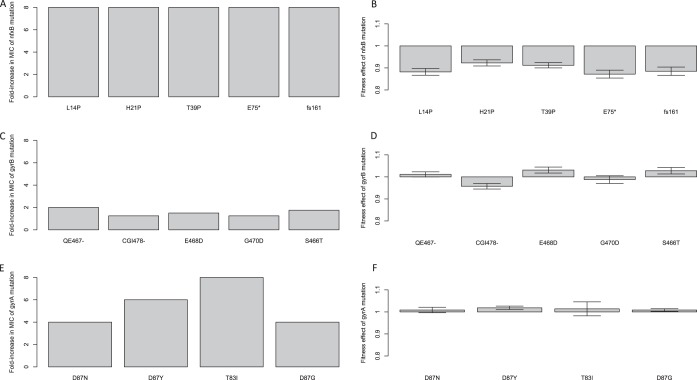
The fold increase in MIC (A, C, and E) and relative fitness effect (B, D, and F) of individual mutations that arose during the course of our selection experiment in *nfxB* (A and B), *gyrB* (C and D), and *gyrA* (E and F). Error bars represent an SE of ±1. MIC values were calculated as medians of results from four trials. Mean relative fitness levels were measured in the absence of antibiotic via direct competitions with a *lacZ*-marked ancestral isolate (PA14) using 1 to 10 replicate measures for each allelic construct. A relative fitness value of less than 1 indicates a cost of resistance in the absence of the antibiotic.

The effects of individual resistance alleles on MIC and fitness are represented in Fig. 4. The most striking result was that mutations to the *nfxB* efflux pump regulator increased resistance 8-fold in every case (Fig. 4A) and were associated with a substantial fitness cost in the absence of drug (Fig. 4B; *t* = −13.1, *df* = 3, *P* < 0.0001). Importantly, there was no tendency for the alleles that evolved under conditions of fluctuating conditions to be less costly than the most common allele (L14P) in the constant environment. Mutations in *gyrA* and *gyrB*, on the other hand, conferred a wider range of MICs and showed no evidence of a cost of resistance (for the *gyrB* constructs, mean fitness, 1.003, SE, ±0.014 [Fig. 4D]; for the *gyrA* constructs, mean fitness, 1.012, SE, ±0.002 SE [Fig. 4F]). It seems likely that the low costs of *gyrA* and *gyrB* mutations arise because they induce conformational changes to DNA gyrases that affect only the binding site of ciprofloxacin (within the quinolone resistance-determining region [QRDR]; 49) and so are not expected to be energetically expensive to the cell. Mutations to *nfxB*, on the other hand, might be expected to be costly *a priori* because they are loss-of-function mutations in a negative regulator that lead to constitutive expression of efflux systems, an energetically expensive activity.

**FIG 5:**
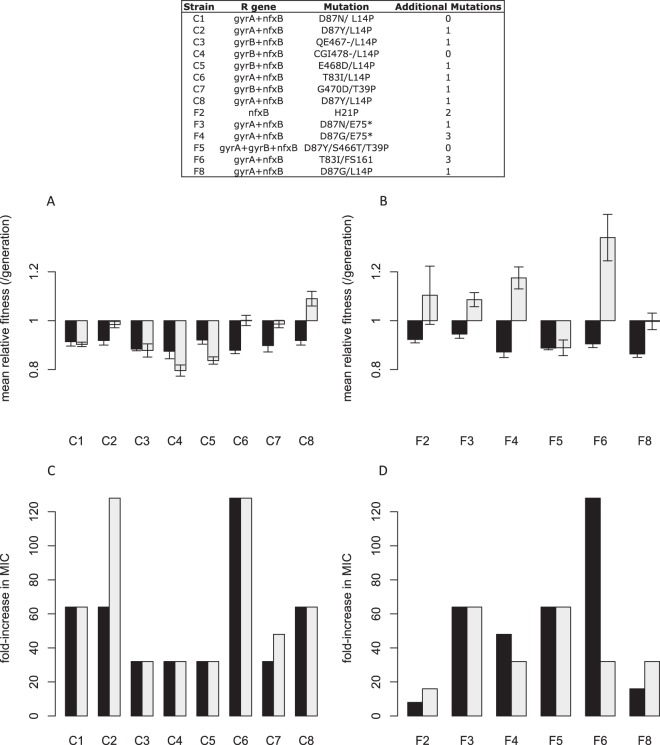
Relative fitness (A and B) and fold increase in MIC (C and D) of either mutant allelic constructs (dark gray) or evolved isolates (light gray) for constant (A and C) and fluctuating (B and D) treatments. The legend provides details on the mutations in the allelic constructs. Error bars represent an SE of ±1. MIC values were calculated as medians of results from four trials. Mean relative fitness was measured in the absence of antibiotic via direct competitions with a *lacZ*-marked ancestral isolate (PA14) using 1 to 10 replicate measures for each allelic construct. A relative fitness value of less than 1 indicates a cost of resistance in the absence of the antibiotic.

The impact on fitness and MIC of combinations of resistance alleles that were fixed by selection in the same lineage is shown in Fig. 5. Three points are worth noting. First, combinations of resistance alleles (dark bars) are costly in both environments as evidenced by mean fitness values whose 95% confidence intervals do not overlap 1.0 (for constant treatment, 0.90 ± 0.03 [mean relative fitness (ω) ± 95% confidence interval calculated from a linear mixed model with genotype as a random effect]; for fluctuating treatment, 0.90 ± 0.01) and indicating that epistasis among resistance mutations does not alleviate costs of resistance. Second, the levels of mean fitness of genotypes containing combinations of resistance alleles in the three lineages for which we did not detect second-site mutations (c1, c4, and f5) were statistically indistinguishable from the levels of mean fitness seen with the corresponding evolved genotypes (light gray bars) (for c1, *t* = 0.539, *df* = 5, *P* = 0.617; for c4, *t* = 2.069, *df* = 5, *P* = 0.088; for f5, *t* = −0.015, *df* = 5, *P* = 0.989) and the median MICs seen with the two groups were statistically indistinguishable also (Fig. 5C and D). These results suggest that the collective effect of second-site mutations in the other lineages is to increase fitness, although some may be neutral or mildly deleterious, having hitchhiked to high frequency alongside other beneficial mutations. Third, for the lineages containing putative second-site mutations, the fitness of reconstructed genotypes with resistance mutations was on average less than that of the corresponding evolved genotypes in the environments of both the constant treatment (Fig. 5A) (χ^2^ test comparing mixed model log likelihoods with or without the fixed effect of genotype class, either resistant or evolved, with replicate population treated as a random effect, *P* = 0.001) and the fluctuating treatment (Fig. 5B) (χ^2^ test performed as described above, *P* < 0.001). These results suggest that second-site mutations tend to improve fitness under both conditions. Notably, fluctuating conditions tend to produce larger fitness improvements associated with second-site mutations, a result confirmed by the significant interaction term in a fully factorial model with treatment (constant versus fluctuating) and genotype (evolved versus constructed) as fixed factors (χ^2^ test performed as described above with and without an interaction term, *P* < 0.001). Taken together, these results provide compelling experimental evidence that second-site mutations are responsible for the evolution of cost-free resistance under fluctuating conditions.

A fourth point, regarding MICs, deserves mention. We could not detect a significant difference in median MIC between reconstructed and evolved genotypes in the constant environment (Fig. 5C) (*W* = 0.20, *P* = 0.655), suggesting that we have identified the majority of resistance mutations fixed in this environment. In the fluctuating environment, in contrast, the differences in median MIC were more variable (Fig. 5D) (*W* = 3.70, *P* = 0.055). The lower median drug MIC seen with reconstructed genotypes for f2 and f8 than with the corresponding evolved genotypes suggested that additional second-site mutations actually confer resistance. In contrast, lines f4 and f6 have higher median MICs for reconstructed genotypes than their evolved counterparts, implying the existence of strong epistasis for resistance between known resistance mutations and second-site mutations. These more complicated interactions between second-site mutations and levels of resistance deserve further investigation.

### A model for the evolution of persistent drug resistance.

While resistance is widely and justifiably recognized as a costly trait, we have shown that the total fitness burden of resistance depends both on the suite of genes that confer resistance and on the ecological conditions in which resistance evolves. Not all mutations are equally costly, and some, such as those to *gyrA* and *gyrB*, may even be cost free. Interestingly, this result could explain why mutations in gyrases are often found at high frequency in many clinical isolates (58): they confer modest levels of resistance without compromising fitness or growth rate. In our experiment, however, these mutations were always accompanied—under both constant and fluctuating conditions—by costly loss-of-function mutations in the *nfxB* efflux pump regulator, suggesting that gyrase mutations are selected because they confer high levels of resistance and not because they are cost free. Importantly, the costs of resistance stemming from *nfxB* mutations or any other costly resistance mutation can be readily circumvented through selection under conditions of fluctuating drug administration. Fluctuating conditions can reduce costs of resistance because the regular periods of relaxed selection that occur when the drug is absent provide ample opportunity for the fixation of second-site mutations that improve fitness but do not compromise resistance. Similar levels of heterogeneity in environmental conditions are not available under conditions of constant drug selection, so selection favors resistance, regardless of the costs.

That said, it is notable that constant drug selection can lead to the occasional evolution of genotypes that both are resistant and maintain modestly high fitness in the absence of drug (see especially population C8 in Fig. 5A). Costs of resistance can therefore sometimes be alleviated by second-site mutations that, in our experiment, probably involved loss of motility and conferred general fitness improvements to the prevailing conditions of growth. The trade-off between resistance and fitness suggests, however, that such genotypes are unlikely to be common. Nevertheless, in large populations, competition among distinct resistance mutations may lead to the evolution of what we might call “high-resistance, cost-reduced” genotypes. These genotypes are likely to be less fit in the absence of drug than those that evolved under conditions of fluctuating treatments, but they are likely to be more resistant and so may be an underappreciated source of antibiotic persistence.

Our results present an admittedly bleak picture for dealing with persistent drug resistance, at least insofar as this one experiment can be taken to be representative of what would happen with other pathogens and other drugs. To the extent that our experiment captures the essential ecology of antibiotic delivery associated with many current therapies such as intermittent administration of oral antibiotics during exacerbations within CF patients, our results suggest that these therapies select for cost-free resistance genotypes. Moreover, our results also suggest that low-cost resistance genotypes may occasionally evolve under conditions of more-persistent drug administration regimes, making the management of antibiotic persistence even more difficult. Taken together, these results suggest that the most effective strategy for dealing with persistence is either to ensure that resistance does not evolve in the first place, perhaps through treatment with multidrug cocktails as is normally practiced in HIV therapy, or to exploit the trade-off between resistance and fitness under no-drug conditions by ensuring that when resistance evolves, it is most likely to do so through mutations that incur a high fitness cost.

## MATERIALS AND METHODS

### Experimental evolution.

A single colony of *P. aeruginosa* strain PA14 was grown overnight in lysogeny broth (LB) (Bacto tryptone [10 g/liter], NaCl [3 g/liter], yeast extract [5 g/liter]). Sixteen populations were founded from this progenitor by adding 20 µl of overnight culture to 1.5 ml of fresh medium (media described below). An aliquot of progenitor was frozen at −80°C in 20% glycerol. Populations were grown in an orbital shaker (150 rpm) at 37°C for 24 h in 24-well plates (Corning, NY, USA). After 24 h, each population was serially propagated by transferring 20 µl of overnight culture to 1.5 ml of fresh medium. Overnight cultures were frozen at −80°C in 20% glycerol every eight transfers, such that approximately 50 generations of adaptation occurred for each freezing cycle.

The selection experiment consisted of growing populations in LB broth supplemented or not supplemented with antibiotic on different schedules. For all the antibiotic treatments, a concentration of 0.8 µg/ml ciprofloxacin was used, because it decreased growth of the sensitive PA14 ancestor to 20% of full growth in LB broth. A concentration of ciprofloxacin that completely inhibited growth was not used because bacterial growth was required to continue our selection experiment. The two treatments differed in the amount of time that the populations were allowed to grow in the absence of the antibiotic. The constant treatment (c) consisted of populations exposed to ciprofloxacin for approximately 50 generations, and the fluctuating treatment (f) consisted of populations grown for two cycles (48 h) in the presence of ciprofloxacin followed by two cycles of growth in the absence of ciprofloxacin for a total of 16 growth cycles, corresponding to a total of 50 generations of growth in the presence of ciprofloxacin. Comparable experimental evolution studies examining the evolution of antibiotic resistance used similar time scales (24-h alternation [59]; total length of ~50 generations [38]).

### Phenotypic analyses.

We isolated a single random colony (here referred to as an “evolved isolate”) from each evolved population at the end of the experiment. For each evolved isolate, the level of resistance was assayed as the ciprofloxacin MIC. Overnight cultures of each evolved isolate were grown in LB broth; 10 µl was diluted into 10-ml LB broth and 100 µl of this dilution was used to inoculate 96-well plates with various concentrations of ciprofloxacin. For each evolved isolate, we assayed growth at 0×, 1×, 2×, 4×, 8×, 16×, 32×, 64×, 128×, 256×, and 512× the ancestral MIC. Log-transformed MICs were used for all statistical analyses.

Relative maximum growth rates of the evolved isolates in the presence and the absence of ciprofloxacin were measured as their maximum growth rates over a 24-h period relative to the ancestral growth rate of PA14 in LB broth. For each assay, 20 µl of overnight culture was added to 180 µl of LB broth and the optical density at 600 nm (OD_600_) was measured by spectrophotometry every 90 min. Growth rates during the exponential phase were then estimated using Gen5 software (Bio-Tek Instruments Inc., Winooski, VT), and the relative growth rate was taken as the mean growth rate of 3 replicates divided by the mean growth rate of the ancestral PA14 genotype grown on the same assay plate. The log-transformed mean relative growth rate was used for all statistical analyses (60).

The relative fitness of each evolved isolate was assayed using a competitive fitness assay against a *lacZ*-marked ancestral isolate. Independent assays verified that the *lacZ*-marked strain did not show a fitness cost in competitions with unmarked PA14 (this experiment and reference 38). Both competitors were grown overnight in LB broth, the competition medium. At time zero, 10 µl of the marked ancestor and 10 µl of the evolved isolate were inoculated into 1.5 ml of fresh medium in a 24-well plate and an aliquot of the mix was frozen at −80°C in glycerol. Following 24 h of growth at 37°C and 150 rpm, a final aliquot was frozen at −80°C in glycerol. Serial dilutions of the initial and final aliquots were grown on solid minimal media plus X-Gal (5-bromo-4-chloro-3-indolyl-β-d-galactopyranoside), allowing us to determine the numbers of blue (ancestral) and white (evolved) individuals at the beginning and end of the competition. The relative fitness, ω, was calculated as follows:

$$\text{ω}={\left[\left(\frac{{\text{white}}_{\text{final}}}{{\text{blue}}_{\text{final}}}\right)-\left(\frac{{\text{white}}_{\text{initial}}}{{\text{blue}}_{\text{initial}}}\right)\right]}^{\left(1/\text{doublings}\right)}$$

The unit for ω is per generation, and "doublings" represents the number of generations occurring between the initial and final measurements.

All statistical analyses were performed in R (61).

### Whole-genome sequencing and analysis.

For whole-genome sequencing, each evolved isolate used in the analysis described above was grown overnight in LB broth, and genomic DNA was extracted using a Promega Wizard genomic DNA purification kit. The Genome Quebec Innovation Centre performed 250-bp paired-end Illumina sequencing using a Mi-Seq platform. Mean coverage across all 16 genotypes was 38-fold (mean, 37.7; range, 28.2 to 49.4) at a mean quality score of 32. The sequence data were aligned to PA14 reference genome number NC_008463.1, mutations were called and annotated using a custom computational pipeline (modified from reference 62), and all mutation alignments were then checked visually using SamTools (ver. 0.1.19).

A subset of mutations was verified by Sanger sequencing of PCR amplicons; for 20 mutations (of 47 mutations identified in 15 evolved isolates), we amplified a 500-to-700-bp PCR product containing the putative mutation and directly sequenced the PCR products (Genome Quebec, Montreal, Canada). All 20 mutations that we examined were successfully verified. Gene location and function were found using the Pseudomonas genome database (63).

### Annotation of gene function.

Annotation of gene function was done using the Pseudocap function as listed on the Pseudomonas genome database (63). We supplemented the “antibiotic resistance and susceptibility” class with additional genes that were known to be involved with fluoroquinolone resistance in *P. aeruginosa*. These included (i) subunits of the targets of ciprofloxacin, DNA gyrase (*gyrA* and *gyrB*) and topoisomerase IV (*parC* and *parE*) (48–50), and (ii) the efflux pump MexAB-OprM and its regulator *mexR* and the efflux pump MexCD-OprJ and its regulator *nfxB* (47). Additionally, the genes with crosshatched notation for the functional class “antibiotic resistance and susceptibility” are genes that have been hypothesized to be involved with fluoroquinolone resistance (*orfN*; reference 38 and this study).

### Variation within *gyrA*, *gyrB*, and *nfxB* in clinical and environmental isolates.

Gene sequences were extracted from the PA14 genome and were BLAST searched against a local database containing the genomes of either 100 CF isolates or 77 environmental isolates (see Table S3 in the supplemental material). Resulting best hits were aligned, and base frequencies were determined for the specific nucleotide positions of interest.

10.1128/mSphere.00158-17.5 TABLE S3  Details of natural isolates (CF, isolated from cystic fibrosis patients; env, isolated from environmental sources) included in variation analysis. Download TABLE S3, XLS file, 0.04 MB. Copyright © 2017 Melnyk et al. 2017 Melnyk et al. This content is distributed under the terms of the Creative Commons Attribution 4.0 International license.

### Allelic constructs.

The *gyrA*, *gyrB*, and *nfxB* mutations were introduced in single or double mutant combinations into the PA14 chromosome using a previously described allelic exchange method (56, 57). Mutations and surrounding sequences were PCR amplified from chromosomal DNA isolated from the evolved bacteria and cloned into a derivative of pAH79, a suicide vector with ampicillin and tetracycline resistance genes and a *sacB* counterselectable marker (57). The plasmids were mobilized into PA14 by triparental mating with the help of pRK2013. Chromosomal integration of the plasmid by homologous recombination was selected using LB agar supplemented with 100 μg/ml nitrofurantoin and 60 μg/ml tetracycline. A second recombination, resulting in loss of plasmid and markerless allelic replacement, was selected by *sacB* counterselection. Tetracycline-resistant clones were plated on LB agar containing 5% sucrose. Successful allelic replacements were distinguished from wild-type revertants by Sanger sequencing (Genome Quebec). Double mutants were constructed by introducing an additional mutation by the same procedure.

### Accession number(s).

Sequencing reads were stored using the Sequence Read Archive database (NCBI), and the accession numbers can be found in Table S4 in the supplemental material.

10.1128/mSphere.00158-17.6 TABLE S4  Accession numbers for isolates sequenced in this study. Download TABLE S4, XLSX file, 0.01 MB. Copyright © 2017 Melnyk et al. 2017 Melnyk et al. This content is distributed under the terms of the Creative Commons Attribution 4.0 International license.
