# Supplementary figures and images for: Evolution of Cost-Free Resistance under Fluctuating Drug Selection in Pseudomonas aeruginosa
Source: mSphere. 2017 Jul 19;2(4):e00158-17. doi: 10.1128/mSphere.00158-17 (PMC5518267; doi:10.1128/mSphere.00158-17)

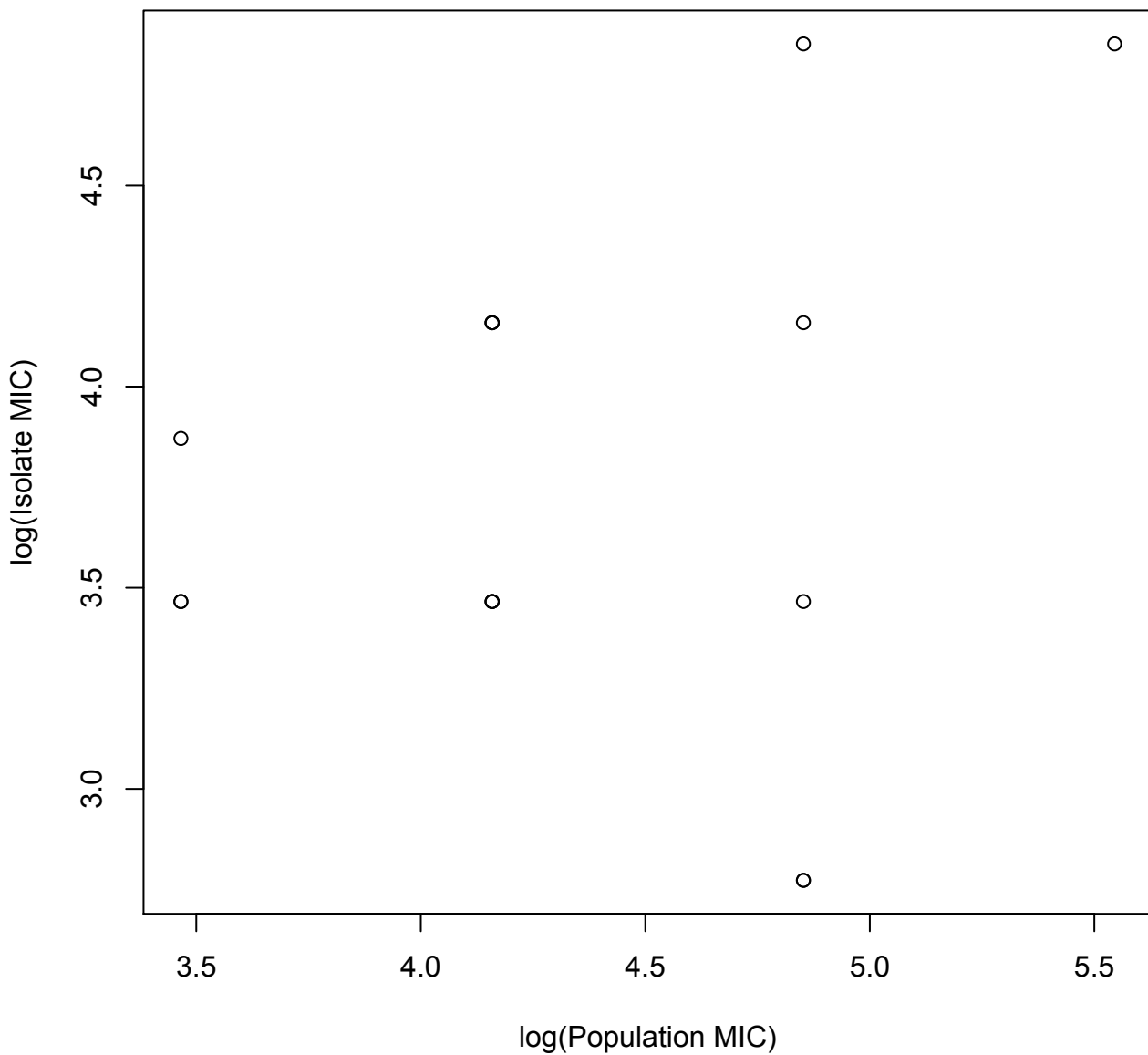

Supplement: FIG S1 [file sph004172317sf1.pdf]

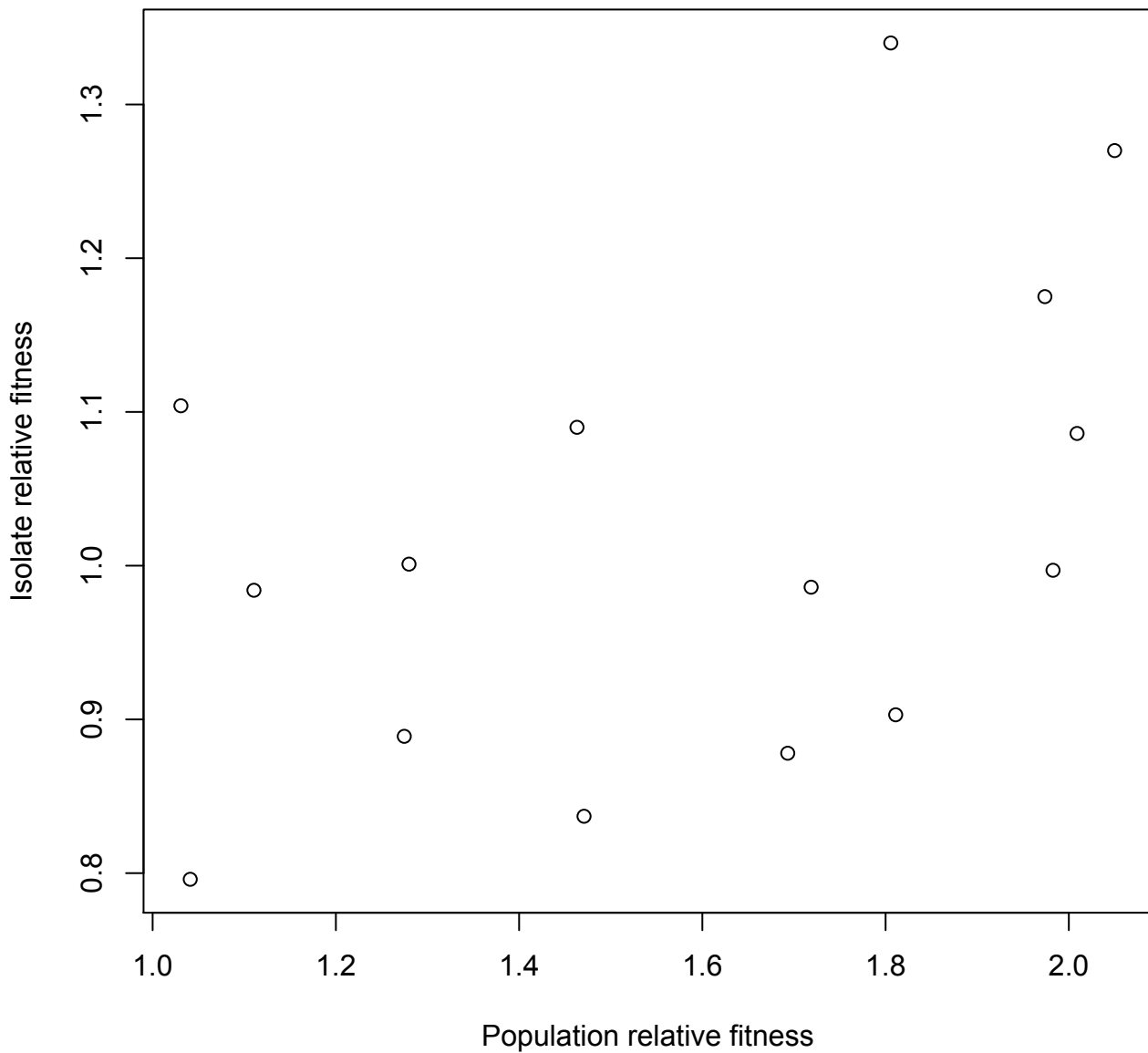

Supplement: FIG S2 [file sph004172317sf2.pdf]
